# Self-Healing Capability of Fiber-Reinforced Cementitious Composites for Recovery of Watertightness and Mechanical Properties

**DOI:** 10.3390/ma7032141

**Published:** 2014-03-13

**Authors:** Tomoya Nishiwaki, Sukmin Kwon, Daisuke Homma, Makoto Yamada, Hirozo Mihashi

**Affiliations:** 1 Department of Architecture and Building Science, Tohoku University, Aoba 6-6-11-1205, Aramaki, Aoba-ku, Sendai 980-8579, Japan; E-Mails: sukminkwon@hjogi.pln.archi.tohoku.ac.jp (S.K.); makotoyamada@hjogi.pln.archi.tohoku.ac.jp (M.Y.); h-mihashi@ab.cyberhome.ne.jp (H.M.); 2 Takenaka Research & Development Institute, Takenaka Corporation, Ohtsuka 1-5-1, Inzai, Chiba 270-1395, Japan; E-Mail: honma.daisuke@takenaka.co.jp

**Keywords:** self-healing, fiber reinforced cementitious composite (FRCC), watertightness, mechanical property, synthetic fiber, steel fiber, bond strength, pull-out test

## Abstract

Various types of fiber reinforced cementitious composites (FRCCs) were experimentally studied to evaluate their self-healing capabilities regarding their watertightness and mechanical properties. Cracks were induced in the FRCC specimens during a tensile loading test, and the specimens were then immersed in static water for self-healing. By water permeability and reloading tests, it was determined that the FRCCs containing synthetic fiber and cracks of width within a certain range (<0.1 mm) exhibited good self-healing capabilities regarding their watertightness. Particularly, the high polarity of the synthetic fiber (polyvinyl alcohol (PVA)) series and hybrid fiber reinforcing (polyethylene (PE) and steel code (SC)) series showed high recovery ratio. Moreover, these series also showed high potential of self-healing of mechanical properties. It was confirmed that recovery of mechanical property could be obtained only in case when crack width was sufficiently narrow, both the visible surface cracks and the very fine cracks around the bridging of the SC fibers. Recovery of the bond strength by filling of the very fine cracks around the bridging fibers enhanced the recovery of the mechanical property.

## 1. Introduction

Significant effort toward the development of a sustainable society has been made in recent times. The maintenance of concrete structures has become one of the most important issues from the ecological point of view. In Japan, several concrete infrastructures built about 50 years ago have deteriorated. Comprehensive maintenance is therefore necessary. However, there has been a decrease in the population of Japan, which has led to the pressing need for labor-saving and cost-effective maintenance systems. Moreover, the functional and/or environmental conditions of some infrastructures such as urban highways and facilities for disposal of radioactive waste make accessibility by engineers for inspection and maintenance difficult or impossible.

The adoption of self-healing methods is one of the most promising approaches to dealing with inherent cracks in concrete structures without human intervention. Many studies have been conducted on the self-healing capability of concrete, especially in the last decade [1–3]. However, a practical solution is still not available. Several studies are ongoing, mainly in Europe, regarding a bacterial approach, e.g., [4,5]. This novel method involves the biological precipitation of calcium carbonate in the cracks through the reaction of CO_2_ produced by bacterial metabolism with Ca(OH)_2_ present in the cement matrix. However, the survival of bacteria has been a challenge, because they are killed during the mixing and setting of concrete, not to talk of long-term survival over the lifetime of concrete structures. Other methods, including the embedment of healing agents (adhesives) contained by capsule and/or vascular approaches, have been investigated [6–11]. In these cases, the pre-maturation of the adhesives before crack initiation and the procedures (mixing and setting of the concrete using brittle pipes and/or capsules) are the challenges that need to be overcome. Moreover, the additional components or devices may be detrimental to the concrete structure. In the vascular method, it is very difficult to construct a network that covers the entire structure like the blood vessels of a human body.

Fiber reinforced cementitious composites (FRCCs) have the greatest potential for the practical achievement of self-healing in concrete. FRCCs are currently commonly used with good results in real applications. Because no special components are required, durability and cost efficiency are assured. Ubiquitous reinforcing fibers in the cement matrix can be used to enhance the self-healing by controlling the crack width and acceleration of CaCO_3_ precipitation [12,13]. Because the healing process does not involve the reaction of the fiber itself, the problem of ensuring the durability of the healing agent is solved. Reinforcement fibers containing high-polarity synthetic composite (e.g., PVA) particularly have strong potentials for self-healing precipitation around the fibers that bridge a crack [14].

In this study, various types of FRCCs were experimentally studied to evaluate their self-healing capabilities regarding their water permeability and mechanical properties.

## 2. Experimental Program

### 2.1. Materials and Mixture Proportions

**Figure 1.**
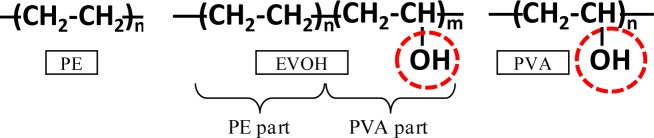
Characteristic part of chemical components.

**Figure 2.**
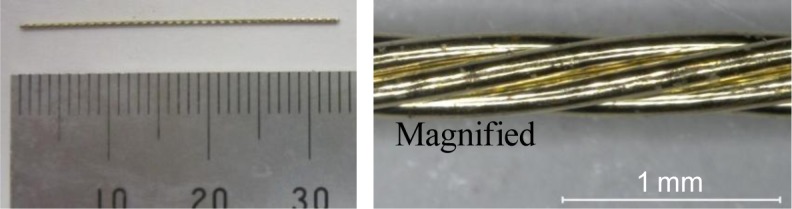
Steel code (SC) fiber.

The mixture proportions of the FRCCs are summarized in Table 1. High-early-strength Portland cement and silica fume were used as the binder. The densities of the cement and silica fume were 3.14 g/cm^3^ and 2.2 g/cm^3^ respectively. The aggregates comprised well-graded and very fine natural silica sand with an average particle size of 180 μm. A polycarboxylate-based superplasticizer was employed. The properties of the employed fibers are presented in Table 2. The characteristic part of the chemical components of the employed synthetic fibers consists of the polarity groups indicated by the circle in Figure 1. PVA has the highest polarity strength owing to the OH radical, whereas PE has no polarity strength. Ethylene vinyl alcohol (EVOH) consists of the PVA and PE parts, hence its moderate polarity strength between those of PVA and PE fibers. On the other hand, steel cord (SC) is composed of five thin steel fibers of diameter 135 μm, which are twisted together as shown in Figure 2. It also has no chemical polarity. Previous studies by the authors have revealed that bridging fibers with high polarity strengths attract calcium ions in cracks, which facilitates the filling of the cracks by the precipitation of CaCO_3_ [13,14].

### 2.2. Preparation of FRCC Specimens

**Figure 3.**
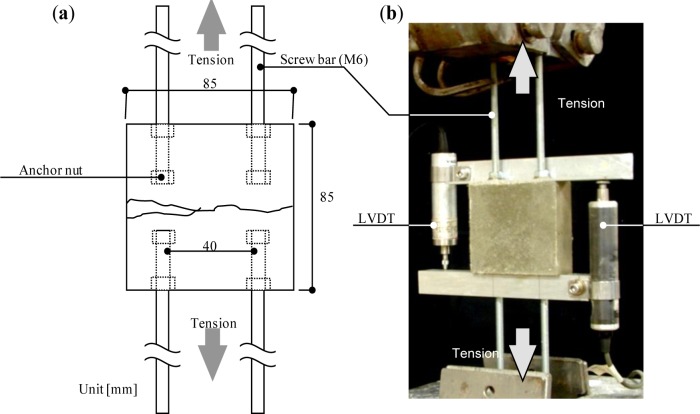
(**a**) Geometry of specimen and (**b**) direction of load during tensile test.

The FRCC mixture was prepared in two liters a Hobart mixer by the following procedure. The cement, silica fume, and sand were first mixed for 1 min. Water and a polycarboxylate-based superplasticizer were then added, and mixing was done for 3 min. Lastly, the fibers were added and mixing was done for another 3 min to achieve proper fiber dispersion for each series. The mixtures were cast in molds. The mold, which measured 85 mm × 85 mm × 30 mm, was prepared with four M6 screw bars, which had anchor nuts at their tips (Figure 3a). The screw bars were embedded in both ends of the specimen to enable holding of the specimen by the testing machine. After de-molding, the specimens were cured in a water tank at 20 °C for 7 days in preparation for testing.

Tension

Tension

### 2.3. Inducement of Cracks during Tensile Loading Test

Direct uniaxial tensile loads were applied to the specimens by a 30 kN capacity load frame under controlled displacement. A quasi-static loading speed of 0.2 mm/min was used. Two external linear variable differential transformers (LVDTs) were attached to the specimen using a gauge length of 85 mm to measure the displacement (Figure 3b).

**Figure 4.**
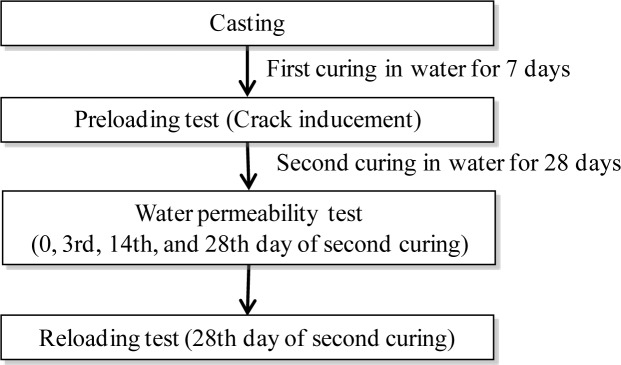
Experimental procedure.

The experimental procedures are summarized in Figure 4. The specimens were de-molded one day after casting and cured in water for 7 days (first curing). The FRCC specimens were then preloaded to induce three levels of cracks (*i.e.*, <0.1 mm, 0.1–0.5 mm and >0.5 mm) in them.

### 2.4. Water Permeability Test and Re-Curing (Second Curing) Method

**Figure 5.**
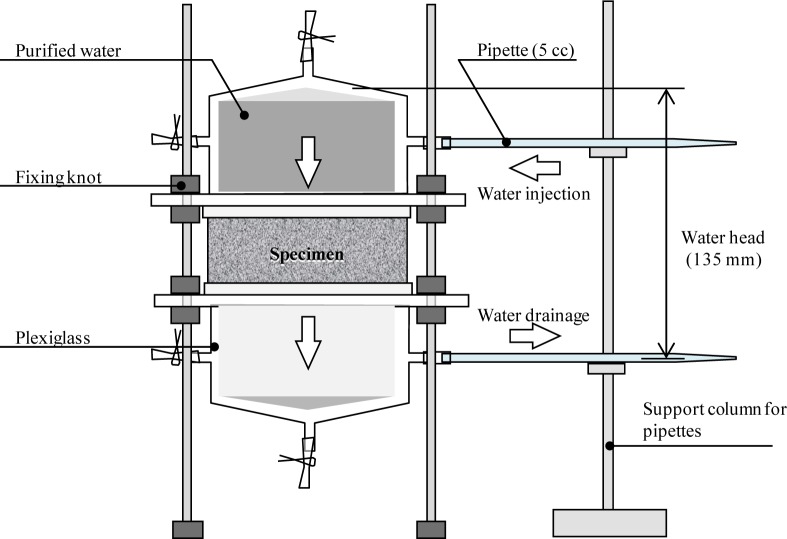
Apparatus of water permeability test.

The first water permeability tests (day 0) were conducted immediately after the tensile test. The apparatus of the water permeability test in this study is shown in Figure 5 [14] (modified after Kishimoto [15]). The coefficient of water permeability was calculated from the volume of water that permeated the specimen. The specimens were cured again in a water tank at 20 °C for 3, 14, and 28 days, respectively (second curing). The water permeability tests were conducted again at the same time after each curing period. After curing for 28 days, the specimens with the induced cracks were reloaded in direct tension test again.

## 3. Results and Discussion

### 3.1. Recovery of Watertightness

Summarized results of the experimental study are shown in Table 3. This table includes the result of watertightness and mechanical properties which will be discussed in the next section.

**Figure 6.**
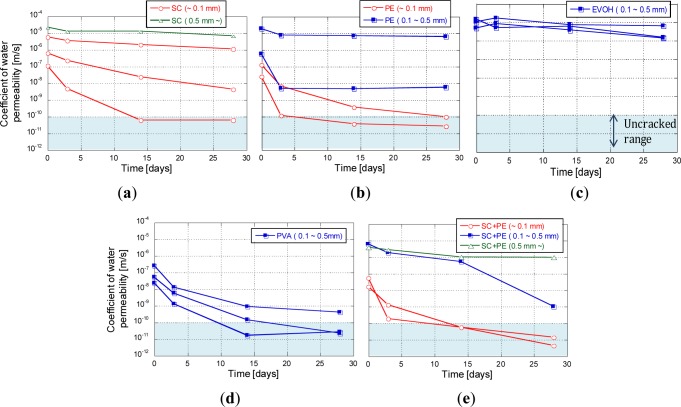
Relationship between time and coefficient of water permeability: (**a**) SC; (**b**) polyethylene (PE); (**c**) ethylene vinyl alcohol (EVOH); (**d**) polyvinyl alcohol (PVA) and (**e**) PE+SC.

**Figure 7.**
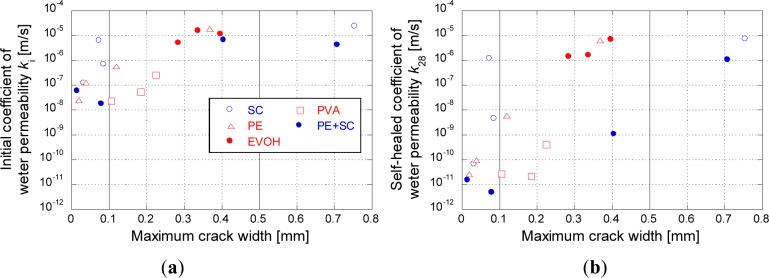
Relationship between the maximum crack width and coefficients of water permeability: (**a**) initial and (**b**) after self-healing.

The variation of the coefficient of water permeability as a function of time during the second curing period is shown in Figure 6. The relationships between the maximum crack width and the initial coefficient of water permeability (*k*_i_) and between the maximum crack width and the coefficient of water permeability after self-healing (*k*_28_) are shown in Figure 7a,b, respectively.

**Figure 8.**
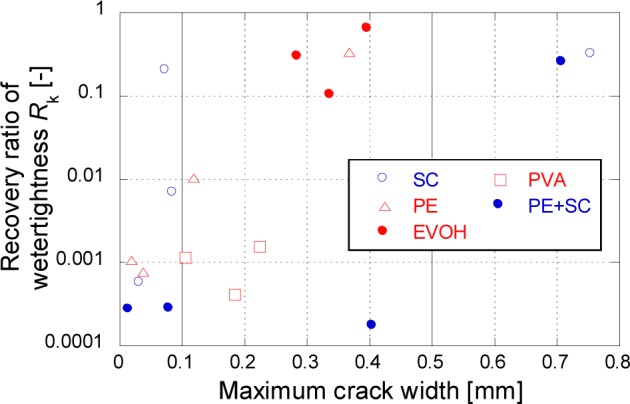
Relationship between the maximum crack width and recovery ratio of watertightness.

For quantifying effect of self-healing on water permeability the recovery ratio was determined by Equitation (1):

$$R_{k}=\frac{k_{28}}{k_{i}}$$ (1)

where *R*_k_ is recovery ratio of watertighness; *k*_i_ is the initial coefficient of water permeability and *k*_28_ is coefficient of water permeability after self-healing. According to this relationship, lower values of *R*_k_ mean large reduction of watertightness of FRCC due to self-healing. The relationship between the maximum crack width and *R*_k_ of all the samples obtained in the present study are shown in Figure 8.

As shown in Figures 6 and 7a, it is confirmed that the wider the crack width, the larger the initial coefficient of water permeability. The recovery of the water permeability was significantly affected by the crack width. The crack widths were measured by a digital microscope. The measured crack widths were divided into three levels, namely, <0.1 mm, 0.1–0.5 mm and >0.5 mm, which are identified by different colors in the legends of Figure 6. As shown in Figure 6, scatters were observed on SC (<0.1 mm) and PE (0.1–0.5 mm) might be caused by difference of the maximum crack width (see Table 3).

With the exception of two specimens in the SC series, for a crack width less than 0.1 mm, the coefficient of water permeability after the second curing was below 1 × 10^−10^ (m/s), which was of the same level as that of the uncracked FRCC (blue-hatched area in the graphs). The maximum crack width strongly affects the capability for self-healing of watertightness. The natural healing that is inherent to cementitious materials, owing to the deposition of calcium carbonate, could be exhibited for a sufficiently small crack width [16]. Only the PVA series shows good recovery even in case of over 0.1 mm crack width and approximately 0.2 mm (Figure 7b) from the point of *k*_28_ view. This recovery was enabled by significant precipitation of calcium carbonate in the cracks due to its high polarity. However, EVOH series did not show any recovery of watertightness, even though EVOH has a moderate polarity which was expected to increase self-healing performance by OH radical as shown in Figure 1. This phenomenon might be related to its brittle behavior which led localization of a crack without multiple cracks after the first crack occurred during the first tensile loading test. Therefore, controlling crack width was very difficult as EVOH series. From the results, polarity of fibers had a potential to improve the self-healing performance, controlling crack width is also very important factors on self-healing capability.

Series of relatively small crack width for exhibited good *R*_k_ (below 0.001) as shown in Figure 8. In this figure, both of PE and SC series show high values of *R*_k_. However, PE + SC series is confirmed to show a good value of *R*_k_ even at 0.4 mm of the maximum crack width which is a larger crack width than those of PVA series (approximately 0.2 mm). This phenomenon might be synergistic effect of two different fibers (PE and SC) for filling up the crack with CaCO_3_ [12] controlling crack propagation. Because of the effect, recovery of water tightness could be possible in PE+SC series.

### 3.2. Recovery of Mechanical Properties

**Figure 9.**
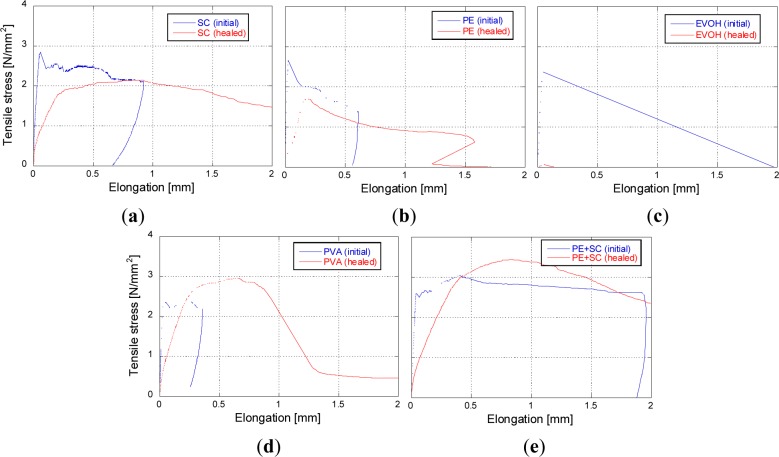
Typical examples of relationship between elongation and tensile stress: (**a**) SC; (**b**) PE; (**c**) EVOH; (**d**) PVA and (**e**) PE+SC.

**Figure 10.**
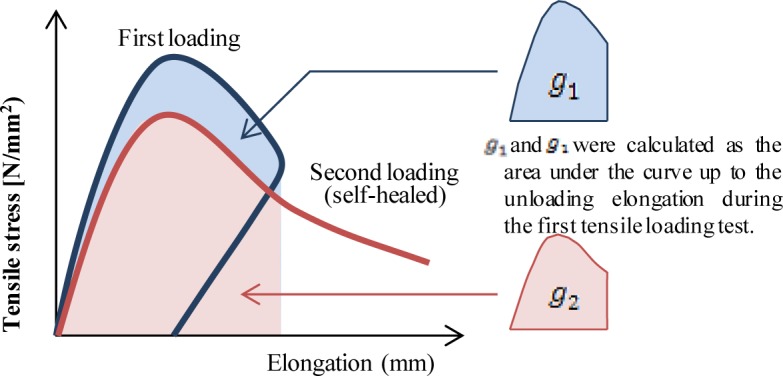
Schematic diagram of relationship between tensile stress and elongation.

After the water permeability tests, the second tensile loading test was conducted to determine the effect of self-healing on the mechanical properties. Typical examples of the relationship between the elongation and the tensile stress are shown in Figure 9. A schematic representations of the relationship between the tensile stress and the elongation during the first and the second tensile loading tests is shown in Figure 10. The recovery ratio of energy absorption (*R_g_*) is defined by the following Equation (2):

$$R_{g}=\frac{g_{2}}{g_{1}}\times 100$$ (2)

where *g*_1_: the energy absorption during the first tensile loading test up to the point of unloading elongation, and *g*_2_: the energy absorption during the second tensile loading test up to the same unloading elongation point of the first tensile loading test. If *R_g_* is higher than 100%, the absorbed energy of the healed specimen would be greater than that of the initial specimen. This means that the mechanical property is completely recovered. However, if *R_g_* is lower than 100%, the mechanical property is partially recovered or not recovered.

**Figure 11.**
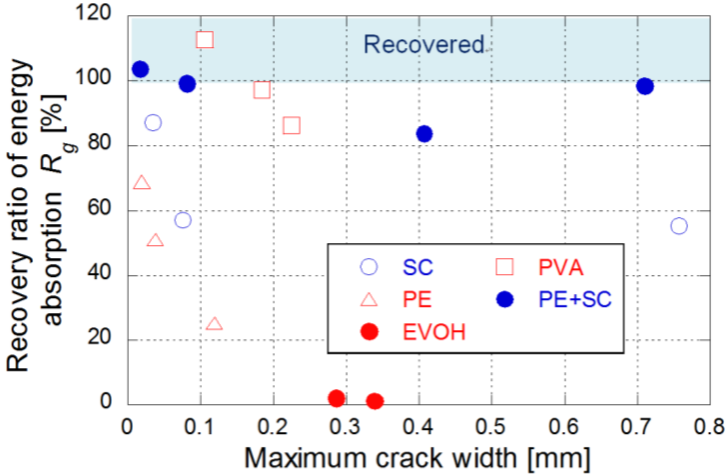
Relationship between recovery ratio of energy absorption (*R_g_*) and maximum crack width.

The relationship between the recovery ratio of energy absorption *R_g_* and the maximum crack width is shown in Figure 11. For sufficiently small maximum crack width (<0.1 mm), the recovery ratio was relatively high. However, the recovery ratio of SC, PE and EVOH series did not exceed 90% of *R_g_*. On the other hand, PE+SC and PVA series showed more than approximately 90% and some specimens show superior to 100% of *R_g_*. Those series occurred in case of the fine width of multiple cracks which led to pseudo strain-hardening behavior. The small crack width might be considered as one of the essential properties in order to recover the mechanical properties. Especially, PE+SC series showed almost 100% of *R_g_* at maximum crack width approximately 0.7 mm. CaCO_3_ fills and closes up the micro cracks and bond strength of SC works for the recovery. Basically, the self-healing mechanism of FRCCs is precipitation of CaCO_3_ and it does not contribute to regain mechanical property [16]. Yang *et al.* [17] was confirmed the mechanical recovery of PVA/matrix by reaction of unhydrated cement and/or fly ash. In this study, although relative higher water binder ratio than their matrix is applied, unhydrated cement still exists in the matrix, which is confirmed by Homma *et al*. [12]. In addition, a pozzolantic reaction of silica fume also expected to positive effect to mechanical recovery.

**Figure 12.**
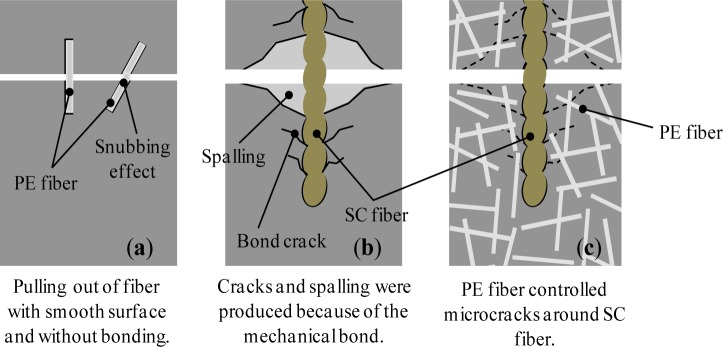
Schematic diagram of pullout behavior of different types of fibers in the matrix.(**a**) Straight fiber without bonding; (**b**) SC fiber and (**c**) SC fiber in FRCC matrix.Z

**Figure 13.**
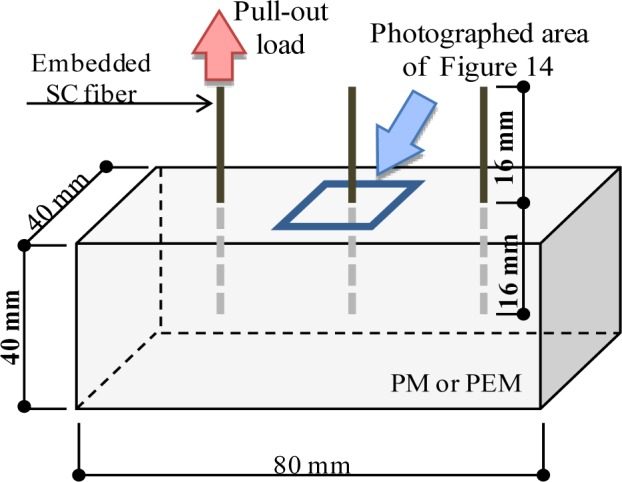
Specimen used for single fiber pullout test.

**Figure 14.**
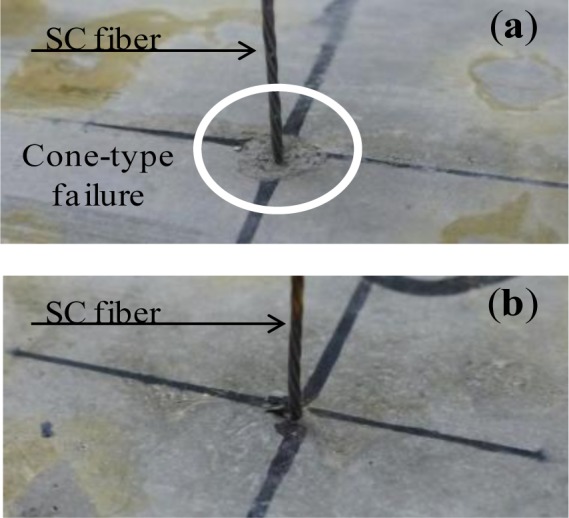
Matrix failure around fiber exit point: (**a**) PM and (**b**) PEM.

Regarding the mechanical recovery of PE+SC series, chemical bond of each fiber is not expected. Therefore, PE+SC series might have different self-healing mechanism from that of PVA series. In addition, PE+SC series is able to be recovered its mechanical property almost up to 100%, even in case that macro crack is occurred (approximately 0.7 mm). This mechanism might be explained by pullout procedure as shown in Figure 12, which illustrates a schematic diagram of the pullout behavior of different types of fibers in the matrix. The PE fiber (microfiber) can control very fine cracks by the bridging effect and snubbing effect [18] (Figure 12a). Whereas SC fiber (macro fiber) is also not expected to form a chemical bond, it forms a strong mechanical bond owing to its unique uneven geometry (Figure 2). An SC fiber can resist visible crack propagation of very fine cracks (Figure 12b). Based on the reinforcement mechanism of a hybrid fiber reinforcing system, that is, long and thick macrofibers and short and thin microfibers are blended, it can be possible to be expected a synergistic effect [19]. By this mechanism, it is expected that the PE+SC series would be able to control both of very fine cracks and visible macro cracks. The PE fiber would support the reinforcement of fine cracks around the SC fiber. On the other hand, the stiff SC fiber can resist macro cracks. Thus, the widths of the cracks are restricted to small values (Figure 12c). This phenomenon was confirmed by the following single fiber pullout test. A schematic diagram of the specimen used for the single fiber pullout test is shown in Figure 13. Fiber pullout specimens with SC fiber inclined at 0 degree were prepared. SC fibers were embedded up to 16 mm (half of the total length) in two different matrices, namely plain matrix (PM) of the same mix proportion shown in Table 1 and matrix reinforced with PE (volume fraction = 0.75%) (PEM). The size of the pullout specimen was 40 mm × 40 mm × 80 mm. After the pullout test, spalling of the matrix was observed (Figure 14). Cone-type failure of the PM was observed at the fiber exit point (Figure 14a). Conversely, very small cracks without spalling were observed around the fiber exit point in the PEM (Figure 14b). These small cracks were filled and closed by self-healing, which was accelerated by the synergistic effect due to PE fiber. In other words, in the case of PE+SC series, small size of cracks around SC fiber was filled by hydration products of unhydrated cement particles, silica fume which activated the pozzolanic reaction, and precipitation of CaCO_3_. Therefore matrix might be densified and performance of mechanical bond was also recovered. From this point of view, mechanical recovery at 0.7 mm of PE+SC series was also obtained by recovery of mechanical bond of SC even though macrocracks were not healed. Self-healing mechanism by the ongoing hydration and aforementioned mechanism of PE+SC series, further experimental researches are required.

## 4. Conclusions

In this study, the self-healing capability of FRCCs was investigated using different types of fibers. Based on the experimental results, the following conclusions are drawn.

(1) If the crack in a FRCC containing synthetic fibers is narrower than 0.1 mm, the watertightness would be recovered regardless the type of fiber as same as watertightness of uncracked FRCC. PVA series, which has polarity, exhibits recovery of the watertightness for crack width of up to approximately 0.2 mm. However, EVOH series, which also has polarity, did not show significant recovered watertightness since the crack width was not well controlled because of the brittle behavior of FRCC. PE+SC series exhibits relatively high recovery ratio of water tightness even in case of the maximum crack widths of 0.4 mm. This is because it generated multiple cracks with fine crack width on the specimens. Recovery ratio of watertightness significantly depends on the crack width. Therefore, controlling crack width is one of important factors in self-healing.
(2) Regarding the mechanical properties of FRCCs after self-healing, a very high recover ratio was confirmed in PVA and PE+SC series. In particular, PE+SC series recovered their mechanical property up to approximately 100% recover ratio even when the crack width was approximately 0.7 mm. This might be because bond crack in the matrix around SC fiber was bridged by PE fiber and the crack width was well controlled. Thus, bond cracks were densifed by self-healing and it linked to the mechanical recovery.

## Figures and Tables

**Table 1.** Mixture proportions of fiber reinforced cementitious composites (FRCCs).

Series	W/B	S/B	SF/B	SP/B	Fiber (vol%)	Number of specimens
SC				–	0.75	4
PE					1.5	4
EVOH	0.45	0.45	0.15	0.009	2.0	3
PVA						3
PE+SC (Hybrid)					0.75+0.75	4

B: binder (cement + silica fume)

**Table 2.** Properties of employed fibers.

Series	Type of Fiber	Density (g/cm^3^)	Tensile strength (N/mm^2^)	Length (mm)	Diameter (μm)
PE	Polyethylene	0.97	2580	6	12
EVOH	Ethylene vinyl alcohol copolymer	1.04	231	5	15
PVA	Polyvinyl alcohol	1.30	1600	6	40
SC	Twisted steel	7.84	2850	32	400

**Table 3.** Summarized results of experimental study.

Series		Max. crack width (mm)	*k*_i_ (m/s)	*k*_28_ (m/s)	*R*_k_ (−)	*g*_1_ (N/mm)	*g*_2_ (N/mm)	*R*_g_ (%)
SC	1	0.035	1.12 × 10^−7^	6.21 × 10^−11^	5.53 × 10^−4^	1.96	1.71	87.4
	2	0.076	5.63 × 10^−6^	1.10 × 10^−6^	1.95 × 10^−1^	5.01	2.85	57.0
	3	0.088	6.26 × 10^−7^	4.23 × 10^−9^	6.76 × 10^−3^	–	–	–
	4	0.757	2.14 × 10^−5^	6.51 × 10^−6^	3.04 × 10^−1^	2.25	1.24	55.3

PE	1	0.019	2.51 × 10^−8^	2.65 × 10^−11^	1.06 × 10^−3^	1.10	0.76	69.1
	2	0.038	1.26 × 10^−7^	9.63 × 10^−11^	7.64 × 10^−4^	1.18	0.60	51.1
	3	0.119	5.57 × 10^−7^	5.84 × 10^−9^	1.05 × 10^−2^	2.26	0.57	25.3
	4	0.368	1.88 × 10^−5^	6.32 × 10^−6^	3.36 × 10^−1^	–	–	–

EVOH	1	0.287	4.61 × 10^−6^	1.33 × 10^−6^	2.87 × 10^−1^	0.06	1.42 × 10^−3^	2.10
	2	0.339	1.44 × 10^−5^	1.46 × 10^−6^	1.01 × 10^−1^	0.06	6.69 × 10^−4^	1.14
	3	0.399	1.03 × 10^−5^	6.41 × 10^−6^	6.25 × 10^−1^	–	–	–

PVA	1	0.106	2.28 × 10^−8^	2.59 × 10^−11^	1.14 × 10^−3^	0.55	0.62	112.9
	2	0.185	5.27 × 10^−8^	2.17 × 10^−11^	4.12 × 10^−4^	0.71	0.69	97.4
	3	0.225	2.56 × 10^−7^	4.03 × 10^−10^	1.57 × 10^−3^	1.22	1.06	86.4

PE+SC (Hybrid)	1	0.017	5.35 × 10^−8^	1.42 × 10^−11^	2.65 × 10^−4^	5.32	5.53	103.8
	2	0.081	1.64 × 10^−8^	4.44 × 10^−12^	2.71 × 10^−4^	5.21	5.18	99.4
	3	0.407	6.03 × 10^−6^	1.02 × 10^−9^	1.69 × 10^−4^	5.78	4.85	83.9
	4	0.710	3.84 × 10^−6^	9.57 × 10^−7^	2.49 × 10^−1^	2.28	2.24	98.6

*k*_i_: initial coefficient of water permeability; *k*_28_: coefficient of water permeability after 28 days self-healing; *R*_k_: recovery ratio of watertightness; *g*_1_: energy absorption at first loading test; *g*_1_: energy absorption at second loading test and *R_g_*: recovery ratio of energy absorption.
